# Stress signalling to fungal stress-activated protein kinase pathways

**DOI:** 10.1111/j.1574-6968.2010.01937.x

**Published:** 2010-03-25

**Authors:** Deborah A Smith, Brian A Morgan, Janet Quinn, Derek Sullivan

**Affiliations:** Institute for Cell and Molecular Biosciences, Faculty of Medical Sciences, Newcastle UniversityNewcastle upon Tyne, UK

**Keywords:** SAPK, model yeasts, *Candida albicans*

## Abstract

The ability of microorganisms to survive and thrive within hostile environments depends on rapid and robust stress responses. Stress-activated protein kinase (SAPK) pathways are important stress-signalling modules found in all eukaryotes, including eukaryotic microorganisms such as fungi. These pathways consist of a SAPK that is activated by phosphorylation through a kinase cascade, and once activated, the SAPK phosphorylates a range of cytoplasmic and nuclear target substrates, which determine the appropriate response. However, despite their conservation in fungi, mechanisms that have evolved to relay stress signals to the SAPK module in different fungi have diverged significantly. Here, we present an overview of the diverse strategies used in the model yeasts *Saccharomyces cerevisiae* and *Schizosaccharomyces pombe*, and the pathogenic fungus *Candida albicans*, to sense and transduce stress signals to their respective SAPKs.

## Introduction

**Fig. 1:**
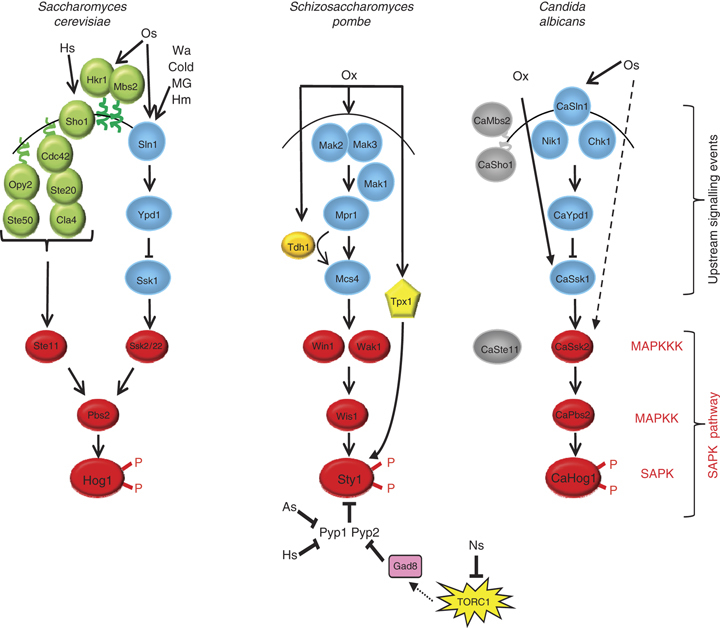
Stress signalling to fungal SAPK pathways. Relay of stress signals to the SAPK pathways in *Saccharomyces cerevisiae*, *Schizosaccharomyces pombe* and *Candida albicans*. Core components of the SAPK pathways are shown in red. Proteins involved in two-component-related signalling are shown in blue and those involved in Sho1-branch signalling in green. *Candida albicans* homologues of proteins that are involved in SAPK activation in *S. cerevisiae*, but not in *C. albicans*, are shown in grey. Only those pathways that have been shown to regulate SAPK phosphorylation are shown, with the exception of the second, uncharacterized, osmotic stress-signalling pathway in *C. albicans*, which is indicated by a dashed line. It is currently unknown how TORC1 and Gad8 regulate Pyp2 levels and hence this is also represented by a dashed line. As, arsenite stress; Hs, heat stress; Os, osmotic stress; Ox, oxidative stress; Wa, weak acid; MG, methylglyoxyl; Hm, heavy metals and Ns, nutrient stress.

Stress-activated protein kinases (SAPKs) are members of the mitogen-activated protein (MAP) kinase family, and are important stress-signalling molecules in all eukaryotic cells (Nikolaou *et al*., 2009). Each SAPK pathway comprises of three tiers of protein kinases: the SAPK itself, a MAP kinase kinase (MAPKK) and a MAPKK kinase (MAPKKK). The MAPKKK phosphorylates and thereby activates the MAPKK, which subsequently phosphorylates the SAPK on conserved threonine and tyrosine residues located in the activation loop of the catalytic domain. This phosphorylation increases the kinase activity and the nuclear translocation of the SAPK, which results in the SAPK-dependent phosphorylation of many substrates including transcription factors, kinases, cell cycle regulators and membrane proteins, thus triggering an appropriate cellular response. The SAPK pathways in the model, but distantly related yeasts, *Saccharomyces cerevisiae* and *Schizosaccharomyces pombe*, and the pathogenic fungus *Candida albicans*, have been well characterized. Each yeast contains a single SAPK and MAPKK, Hog1 and Pbs2 in *S. cerevisiae* (Brewster *et al*., 1993), CaHog1 and CaPbs2 in *C. albicans* (San Jose *et al*., 1996; Arana *et al*., 2005) and Sty1 (also known as Spc1 or Phh1) and Wis1 in *S. pombe* (Millar *et al*., 1995) (Fig. 1). Interestingly, however, the number of MAPKKKs that relay stress signals to the fungal MAPKKs varies. For example, the *S. cerevisiae* Hog1 pathway is regulated by three MAPKKKs, the highly homologous Ssk2 and Ssk22 kinases, and Ste11 (Maeda *et al*., 1995; Posas & Saito, 1997), the *S. pombe* Sty1 pathway is regulated by two MAPKKKs, Wak1 (also known as Wis4) and Win1 (Samejima *et al*., 1997, 1998; Shieh *et al*., 1997), whereas the *C. albicans* CaHog1 kinase is regulated by a single MAPKKK, CaSsk2 (Cheetham *et al*., 2007) (Fig. 1).

The SAPKs are among the most evolutionarily conserved stress-signalling proteins in fungi (Nikolaou *et al*., 2009). It is remarkable, therefore, that the mechanisms that have evolved to relay stress signals to the SAPK pathways in the model yeasts *S. cerevisiae* and *S. pombe* have diverged significantly. Furthermore, there is growing evidence that the role and regulation of the *C. albicans* SAPK pathway differs from that in these model yeasts. However, despite such differences, one universal mechanism by which stress signals are sensed and relayed to the SAPK modules in these fungi involves a two-component signal transduction pathway. Two-component pathways, consisting of a His–Asp phosphorelay system, are widely used in bacteria to respond to environmental signals (Egger *et al*., 1997). Upon activation, a sensor histidine kinase is autophosphorylated on a conserved histidine residue, which in turn phosphorylates a conserved aspartate residue in the receiver domain of a response regulator protein, which then triggers an appropriate response. However, a more complex multistep phosphorelay involving three components is prevalent in fungal systems that comprise a hybrid sensor histidine kinase, containing both kinase and receiver domains, an intermediary phosphorelay protein and a response regulator protein containing a receiver domain. Although two-component-mediated signal transduction regulates the SAPK pathways in *S. cerevisiae*, *S. pombe* and *C. albicans*, their precise roles in stress signalling have diverged significantly. Here, we discuss the different roles of two-component regulation of the SAPKs in these three fungi, together with an overview of the many identified two-component independent mechanisms of SAPK activation. A summary of the stress-signalling mechanisms in the *S. cerevisiae*, *S. pombe* and *C. albicans* SAPK pathways is shown (Fig. 1).

## Regulation of the *S. cerevisiae* Hog1 SAPK

The first SAPK pathway to be identified was the high-osmolarity glycerol (Hog1) pathway of *S. cerevisiae* (Brewster *et al*., 1993) and, as its name suggests, was originally described as an osmosensing pathway. However, subsequent studies revealed that Hog1 is also activated in response to a variety of stress stimuli including oxidative stress (Bilsland *et al*., 2004), heat stress (Winkler *et al*., 2002), cold stress (Hayashi & Maeda, 2006; Panadero *et al*., 2006), citric acid (Lawrence *et al*., 2004), arsenite (Thorsen *et al*., 2006), methylglyoxal (Maeta *et al*., 2005) and weak acid stress (Mollapour & Piper, 2006) (Table 1).

**Table 1:** Stresses that have been shown to activate the SAPKs pathways in *Saccharomyces cerevisiae*, *Schizosaccharomyces pombe* and *Candida albicans*

	Oxidative	Osmotic	Heavy metals	Heat	Cold	Methylglyoxyl	Weak acid	Carbon starvation	Nitrogen limitation	Pressure stress	UV light	Glucose	Cationic peptides
*S. cerevisiae* Hog1	Yes	Yes	Yes	Yes	Yes	Yes	Yes	ND	ND	ND	ND	ND	ND
*S. pombe* Sty1	Yes	Yes	Yes	Yes	Yes	Yes	ND	Yes	Yes	Yes	Yes	ND	ND
*C. albicans* Hog1	Yes	Yes	Yes	No	No	ND	ND	ND	ND	ND	ND	Yes	Yes

Relevant references are cited in the main body text.

ND, not determined.

Osmotic stress activation of Hog1 is one of the best-described stress-signalling pathways (for example, see Hohmann, 2002 as one of many reviews), and hence will only be described briefly here. Two functionally redundant pathways, the Sln1 two-component signalling pathway and a pathway that contains the SH3-domain-containing Sho1 transmembrane protein, converge at the MAPKK Pbs2 to relay osmotic stress signals to Hog1. In the first pathway, loss of turgor pressure, induced by high osmolarity, inactivates the transmembrane histidine kinase Sln1 and thus halts phosphorelay through the phosphorelay protein Ypd1, leading to a rapid dephosphorylation of the Ssk1 response regulator (Posas *et al*., 1996). It has been proposed that disruption of the stable Ypd1–Ssk1∼P complex under increasing concentrations of osmotic stress renders Ssk1 susceptible to dephosphorylation (Kaserer *et al*., 2009). Dephosphorylated Ssk1 has been proposed to activate the MAPKKKs Ssk2/22 in a two-step mechanism, in which binding of unphosphorylated Ssk1 changes the conformation of Ssk2/22 so that the N-terminal autoinhibitory domain of these kinases no longer interacts with the kinase catalytic site. This then allows Ssk2/22 to autophosphorylate the activating phosphorylation sites in the catalytic domain (Posas & Saito, 1998). Indeed, constitutive activation of Ssk2 is observed when Ssk1 cannot be phosphorylated at the conserved Asp554 phosphorylation site or when mutations of Ssk1 prevent interactions with Ypd1 (Horie *et al*., 2008). *Saccharomyces cerevisiae* contains a second response regulator Skn7, in addition to Ssk1. Skn7 is a transcription factor that regulates the expression of both cell wall genes and genes involved in the oxidative stress response. Interestingly, two-component-mediated phosphorylation of Skn7 is only required for the cell wall functions of this transcription factor (Li *et al*., 2002).

In the second pathway, the Stell MAPKKK phosphorylates Pbs2 when stimulated by osmotic stress signals received from the Sho1 branch of the Hog1 pathway (Tatebayashi *et al*., 2006). Many proteins have been implicated in the transmission of the osmosignal through this branch from Sho1 to Ste11-Pbs2, including Cdc42, Ste20, Cla4, Ste50 and Opy2 (Tatebayashi *et al*., 2006; Wu *et al*., 2006). The current proposed model for activation via this pathway involves two crucial steps: first, the Ste11/Ste50 complex is activated by the Ste20/Cla4 kinases, and second Pbs2, is then activated by Ste11 (Tatebayashi *et al*., 2006). Sho1 was originally thought to function as the osmosensor at the top of the pathway (Maeda *et al*., 1995). However, recent findings have revealed that two transmembrane mucins, Msb2 and Hkr1, may be the osmosensors in the Sho1 branch. Msb2 and Hkr1 interact with Sho1 and localize to similar discreet areas of the membrane as Sho1 in response to osmotic stress. However, they appear to activate the Sho1 pathway via two different mechanisms: in the first, Hkr1 or Msb2 associate with Sho1 via their transmembrane domains to generate an intracellular signal while the second is independent of Sho1 and Hkr1, but requires the cytoplasmic region of Msb2 (Tatebayashi *et al*., 2007). Interestingly, while Msb2 and many of the Sho1 branch components also participate in the filamentous growth MAPK pathway, Hkr1 plays a specific role in Hog1 signalling (Pitoniak *et al*., 2009).

In contrast to osmo-stress-signalling mechanisms, much less is known regarding the relay of other stress signals to Hog1. Hog1 is phosphorylated upon exposure to cold stress in a two-component pathway-dependent manner. Cold stress is thought to bring about a reversible rigidification of the plasma membrane, suggesting that Sln1 can sense changes in the fluidity of the plasma membrane (Hayashi & Maeda, 2006; Panadero *et al*., 2006). The two-component pathway has also been implicated in mediating stress signals to Hog1 in response to citric acid stress (Lawrence *et al*., 2004), arsenite (Thorsen *et al*., 2006), weak acid stress (Mollapour & Piper, 2006) and methylglyoxal (Aguilera *et al*., 2005; Maeta *et al*., 2005). Conversely, activation of the Hog1 SAPK in response to heat stress is dependent on the Sho1 branch of the pathway, and not two-component signalling (Winkler *et al*., 2002). It is unclear what upstream signalling events relay oxidative stress signals to Hog1, but cells lacking either Hog1, Pbs2, Sho1 or proteins involved in two-component signalling display increased sensitivity to oxidative stress agents (Singh, 2000; Bilsland *et al*., 2004).

## Regulation of the *S. pombe* Sty1 SAPK

It has long been recognized that the Sty1 SAPK in *S. pombe* is robustly activated by a diverse range of stress conditions (Table 1) including osmotic stress (Millar *et al*., 1995), oxidative stress and heat shock (Degols *et al*., 1996), nitrogen limitation (Shiozaki & Russell, 1996), carbon starvation (Morigasaki *et al*., 2008), UV light (Degols & Russell, 1997), methylglyoxal (Takatsume *et al*., 2006), cold stress (Soto *et al*., 2002), arsenite (Rodriguez-Gabriel & Russell, 2005) and pressure stress (George *et al*., 2007).

*Schizosaccharomyces pombe* contains a two-component system that is architecturally similar to the Sln1–Ypd1–Ssk1 pathway in *S*. *cerevisiae* (see Fig. 1). However, strikingly, the two-component signalling system in fission yeast functions to relay H_2_O_2_, but not osmotic, stress signals to the Sty1 SAPK pathway. Similar to the Ssk1-MAPKKK regulation in *S*. *cerevisiae*, the Mcs4 response regulator, which is a functional homologue of Ssk1, directly interacts with the Wak1 MAPKKK to regulate the Sty1 pathway (Buck *et al*., 2001). Whether Mcs4 activates Wak1 in a manner analogous to that proposed for Ssk1 regulation of Ssk2 in *S. cerevisiae* (Posas & Saito, 1998) is, however, unknown. Similar to the Ypd1 regulation of Ssk1 phosphorylation in *S*. *cerevisiae*, *S*. *pombe* also contains a protein Mpr1, which is predicted to act in phosphorelay to Mcs4 (Nguyen *et al*., 2000). However, in contrast to *S*. *cerevisiae*, there are three histidine kinases in fission yeast: two highly related proteins Mak2 and Mak3 and a third Mak1 (Buck *et al*., 2001). Mutation of the conserved aspartic acid phosphorylation site on Mcs4 (Asp412), or the conserved histidine phosphorylation site on Mpr1 (His221), or deletion of either *mak2*^+^ or *mak3*^+^ (but not *mak1*^+^), significantly impairs the relay of H_2_O_2_ signals to the Sty1 SAPK (Nguyen *et al*., 2000; Buck *et al*., 2001; Quinn *et al*., 2002). Conversely, Mak1 appears to play an inhibitory role in oxidative stress signalling to Sty1 at low concentrations of peroxide stress (Quinn *et al*., 2002). Hence, despite the architectural similarity, the two-component systems connected to the SAPK pathways in *S. pombe* and *S. cerevisiae* are responsible for sensing different stress stimuli. Much less is known regarding the precise mechanism underlying two-component signalling of stress signals to Sty1 in *S. pombe*. However, the *S. pombe* Mak2 and Mak3 histidine kinases have potential oxidative stress-sensing domains that are lacking in Sln1. For example, both Mak2 and Mak3 contain PAS and GAF domains that are located adjacent to the histidine kinase domain. PAS domains are evolutionarily conserved motifs that are implicated in signal transduction, including redox signalling (Taylor & Zhulin, 1999). GAF domains are structurally similar, but unrelated in amino acid sequence, to PAS domains (Hurley, 2003). GAF domains have been shown in many instances to bind cyclic nucleotides, although recent work has revealed that a redox-sensing GAF domain in the DosS histidine kinase in *Mycobacterium tuberculosis* binds haem (Cho *et al*., 2009). It remains to be demonstrated whether the PAS and GAF domains in Mak2 and Mak3 have peroxide-sensing functions. Remarkably, however, a recent study revealed that the glycolytic enzyme GAPDH (Tdh1) is required to promote oxidative stress signalling through the two-component pathway to Sty1 (Morigasaki *et al*., 2008). Tdh1 was unexpectedly found to be present in a complex with the MAPKKKs Wak1 and Win1, and the Mcs4 response regulator. Furthermore, upon exposure to oxidative stress, Tdh1 is transiently oxidized at the redox-sensitive residue Cys152 and this promotes its interaction with Mcs4. Significantly, a point mutation at Cys152 decreased the interaction between Mcs4 and Mpr1, thus leading to a defect in peroxide signalling through the two-component phosphorelay (Morigasaki *et al*., 2008).

Peroxide-induced activation of Sty1 also requires the thioredoxin peroxidase enzyme Tpx1, in a Wis1-dependent mechanism, that acts downstream of the two-component pathway (Veal *et al*., 2004). Moreover, unlike the two-component pathway, which is more important for the activation of Sty1 at low, but not high levels of peroxide stress (Quinn *et al*., 2002), Tpx1 regulates Sty1 activation in response to both high and low levels of H_2_O_2_. The mechanism of regulation of Sty1 by Tpx1 is currently unclear, but upon peroxide stress, intermolecular disulphide bonds are formed between conserved cysteine residues in Sty1 and Tpx1, suggestive of a direct role. These findings illustrate that the antioxidant enzyme Tpx1 also has peroxide sensing and signalling functions in *S. pombe* (Veal *et al*., 2004).

In contrast to peroxide stress, heat stress-induced activation of the Sty1 SAPK occurs by a different mechanism. For instance, in contrast to the strong activation of the Wis1 MAPKK following osmotic or oxidative stresses, the major regulatory event upon heat shock occurs downstream of Sty1 phosphorylation; the interaction between Sty1 and Pyp1, one of the two tyrosine phosphatases that dephosphorylates Tyr173 and inactivates Sty1, is inhibited (Shiozaki *et al*., 1998; Nguyen & Shiozaki, 1999). Thus, Sty1 activation in response to heat stress is mediated, at least in part, by inhibition of a phosphatase. However, this activation is transient due to the subsequent action of the type 2C serine/threonine phosphatases, Ptc1 and Ptc3, which dephosphorylate Thr171 of Sty1 upon exposure to heat (Nguyen & Shiozaki, 1999). Arsenite stress-induced activation of Sty1 has also been shown recently to be partly mediated by inhibition of Pyp1 (Rodriguez-Gabriel & Russell, 2005). Interestingly, Sty1 activation in response to nutrient limitation also involves tyrosine phosphatase inhibition. In particular, changes in nutritional status alter signalling through target of the rapamycin complex (TORC) and a downstream effector, the AGC kinase Gad8, resulting in the downregulation of another tyrosine phosphatase, Pyp2, that targets Sty1 (Petersen & Nurse, 2007; Hartmuth & Petersen, 2009).

Much less is known about the relay of other stress stimuli to the Sty1 SAPK. However, it has been noted that, in contrast to mutation of the phosphoaspartate residue on Msc4 that specifically inhibits peroxide-induced activation of Sty1, deletion of *mcs4*^+^ prevents efficient activation of Sty1 in response to a variety of stresses (Shieh *et al*., 1997; George *et al*., 2007). Whether Mcs4 actually provides a platform to receive diverse stress signals or whether it functions as a scaffolding protein to maintain a functional SAPK module is unclear.

## Regulation of the *C. albicans* Hog1 SAPK

Although less well studied than the analogous pathways in the model yeasts *S. cerevisiae* and *S. pombe*, it is evident that the *C. albicans* CaHog1 SAPK also responds to diverse stress conditions (Table 1). For example, CaHog1 is activated in response to osmotic and oxidative stresses (Alonso-Monge *et al*., 2003; Smith *et al*., 2004), heavy metals, various drugs (Smith *et al*., 2004), cationic peptides (Vylkova *et al*., 2007) and increased glucose levels (Rodaki *et al*., 2009). Interestingly, in contrast to the SAPK pathways in *S. cerevisiae* and *S. pombe*, which are activated following exposure of cells to temperatures of 37 °C or above, CaHog1 is not. In stark contrast, the basal level of Hog1-phosphorylation is reduced following such temperature increases (Smith *et al*., 2004). However, *C. albicans* has evolved to thrive in warm-blooded mammalian hosts, and hence this may reflect adaptation of this pathway not to perceive temperatures of 37 °C as a stress. Moreover, consistent with the mounting evidence that stress responses are crucial for *C. albicans* survival within the host, cells lacking *CaHOG1* display significantly attenuated virulence in a mouse model of disease (Alonso-Monge *et al*., 1999).

Two-component proteins, related to those in *S. cerevisiae* and *S. pombe*, have also been identified in *C*. *albicans* (see Kruppa & Calderone, 2006, for a review). Interestingly, *C. albicans* contains three distinct histidine kinases, CaSln1, Chk1 and Nik1/Cos1, which appear to consist of a marriage of domains in histidine kinases from three distantly related fungi (Fig. 1). For example, CaSln1 is homologous to Sln1 in *S. cerevisiae* (Nagahashi *et al*., 1998), Chk1 is a close homologue of the *S*. *pombe* oxidative stress sensors Mak2 and Mak3 (Calera & Calderone, 1999a, b) and Nik1 is most similar to the osmosensing Nik-1/COS kinase in *Neurospora crassa* (Alex *et al*., 1998). Analogous to *S*. *cerevisiae* and *S*. *pombe*, a single potential phosphorelay protein (CaYpd1) and two response regulators (CaSsk1, CaSkn7) have also been identified (Calera & Calderone, 1999b; Calera *et al*., 2000; Singh *et al*., 2004). Importantly, similar to CaHog1, mutation of two-component genes revealed that two-component signal transduction is required for *C. albicans* virulence (reviewed in Kruppa & Calderone, 2006). Interestingly, although *C. albicans* is more closely related to *S. cerevisiae* than *S. pombe*, CaHog1 phosphorylation is drastically reduced in cells lacking the CaSsk1 response regulator, in response to oxidative, but not osmotic stress agents (Chauhan *et al*., 2003). Furthermore, *Cassk1*Δ cells are more sensitive to oxidative, but not osmotic stress. Intriguingly, a point mutant of the predicted phosphoaspartate residue D556 in the response regulator domain of Ssk1 to asparagine is more sensitive to oxidative stress than *Cassk1*Δ cells. Furthermore, although Hog1 is phosphorylated in response to peroxide stress in cells expressing the CaSsk1D556N mutant protein, in contrast to wild-type cells, Hog1 fails to translocate to the nucleus (Menon *et al*., 2006). Chk1 is the most likely candidate for a potential peroxide-sensing histidine kinase in *C. albicans* as this shows significant similarity to the peroxide-sensing histidine kinases, Mak2 and Mak3, in *S. pombe* (Buck *et al*., 2001). However, although *chk1*Δ cells do display increased sensitivity to oxidative stress (Li *et al*., 2004), deletion of *CHK1* does not impair oxidative stress-induced activation of Hog1 (Li *et al*., 2004; Roman *et al*., 2005). Furthermore, wild-type levels of oxidative stress-induced Hog1 phosphorylation were also observed in double *chk1*Δ*Casln1*Δ and *chk*Δ*nik1*Δ mutants (Roman *et al*., 2005). Hence, it remains unclear how oxidative stress signals are relayed to the CaSsk1 response regulator. Indeed, as Hog1 activation is seen in cells expressing CaSsk1 lacking the predicted phosphoaspartate residue D556, this suggests that CaSsk1 may regulate peroxide-induced phosphorylation of CaHog1 independent of two-component-mediated phosphorylation.

Similar to studies of Sln1 in *S. cerevisiae*, high basal levels of CaHog1 phosphorylation are observed in *Casln1*Δ cells (Roman *et al*., 2005). However, in contrast to *S. cerevisiae*, loss of CaSln1 function is not lethal in *C. albicans* (Nagahashi *et al*., 1998). Nonetheless, the high basal level of CaHog1 phosphorylation in cells lacking *CaSLN1*, and the high level of sequence conservation between CaSln1 and Sln1 in *S. cerevisiae*, suggests that the CaSln1–CaYpd1–CaSsk1 pathway relays osmotic stress signals to the SAPK module. In addition, homologues of all the identified components of the Sho1 osmotic stress-signalling branch in *S. cerevisiae* are present in the *C. albicans* genome database (http://www.candidagenome.org). However, in stark contrast to *S. cerevisiae* (Maeda *et al*., 1995), creation of a double *Cassk1*Δ*Casho1*Δ mutant does not result in impaired resistance to osmotic stress or prevent osmotic stress-induced activation of CaHog1 (Roman *et al*., 2005). Interestingly, a recent study revealed that deletion of *CaMSB2*, encoding the CaMsb2 mucin, only caused osmotic stress sensitivity in cells that also lacked *CaSSK1* and *CaSHO1*, although no defect in osmotic stress-induced activation of Hog1 was observed (Roman *et al*., 2009). Indeed, as osmotic stress-induced activation of CaHog1 is entirely dependent on the MAPKKK CaSsk2, the Sho1 branch in *C. albicans* (which is predicted to relay signals to the CaSte11 MAPKKK) may play no role in CaHog1 regulation (Cheetham *et al*., 2007). Significantly, the N-terminal region of the ScPbs2 MAPKK, which receives signals from the MAPKKKs Ste11, Ssk2 and Ssk22 in *S. cerevisiae* has greatly diverged in the CaPbs2 kinase. However, expression of a Pbs2 chimera in which the N-terminal regulatory domain of ScPbs2 was fused to the CaPbs2 C-terminal kinase domain still failed to stimulate CaSho1-CaSte11-dependent signalling to CaHog1 (Cheetham *et al*., 2007). Taken together, the current data indicate that, in addition to the CaSln1 phosphorelay system, a novel osmotic stress-sensing pathway is present in *C. albicans*, and moreover, that both pathways converge on the CaSsk2 MAPKKK. How CaSsk2 is activated by such pathways, however, remains to be determined.

## Summary

The SAPKs predominate as highly conserved signalling molecules found throughout the fungal kingdom. However, in contrast, the sensors and signal transducers that lie upstream of these SAPKs are much less well conserved (Nikolaou *et al*., 2009). Indeed, such differences are exemplified by the different sensing mechanisms used by the model yeasts *S. cerevisiae*, and *S. pombe*, and the pathogenic fungus *C. albicans*, as highlighted in this review. For example, in *S. cerevisiae* osmotic stress signalling to Hog1 occurs via the functionally redundant Sho1 and Sln1 branches. However, in *C. albicans*, the Sho1 branch does not appear to relay osmotic stress signals to CaHog1, and in *S. pombe*, no functional Sho1 or Sln1 homologues have been found. Indeed, despite many studies of the Sty1 pathway in *S. pombe*, it is entirely unknown how osmotic stress signals are relayed to the Sty1 SAPK. However, it is tempting to speculate that such an osmotic stress-sensing mechanism is also present in *C. albicans*, as clearly there is an unidentified additional pathway(s) that relays osmotic stress signals to CaHog1 in parallel with the Sln1 branch. In addition, it is intriguing that the histidine kinases sense osmotic stress in *S. cerevisiae* yet oxidative stress in *S. pombe*. Such differences may reflect an adaptation to the specific environmental niches occupied by these fungi, as suggested by a recent study indicating that upstream components of fungal stress response pathways have evolved rapidly to allow survival in diverse environments (Nikolaou *et al*., 2009).
